# Predictive factors for the outcomes of Graves’ disease patients with radioactive iodine (^131^I) treatment

**DOI:** 10.1042/BSR20191609

**Published:** 2020-01-03

**Authors:** Yu-Zhuo Xing, Kun Zhang, Gang Jin

**Affiliations:** Department of Nuclear Medicine, The Second Affiliated Hospital, Harbin Medical University, Harbin 150081, Heilongjiang, P.R. China

**Keywords:** graves’ disease, radioactive iodine, radioiodine therapy, thyroid radioactive iodine uptake

## Abstract

Although radioactive iodine (^131^I) treatment (RIT) is recommended as the preferred option for patients with Graves’ disease (GD), the predictive factors for its clinical outcomes are still unclear. In the present study, we aim to investigate the factors influencing the success rate of RIT treatment on primary GD with a calculated dose approach. The thyroid function (hyperthyroidism, euthyroidism or hypothyroidism) was evaluated at least 1 year after RIT, and its relationship with presenting clinical characteristics and pre-RIT parameters was analyzed in 45 patients retrospectively. After RIT, the remission rate was 62.2%, including 13 euthyroidism cases (28.9%) and 15 hypothyroidism cases (33.3%). We found no significant association between the types of thyroid function and age, gender, the 3-h radioactive iodine uptake (RAIU) prior to RIT, or radioactive iodine (^131^I) dosage. However, a variable 24-h RAIU > 46.31% was found associated with the success rate of RIT. The present study implied that a calculated dose approach for GD is effective, but high failure rates are expected in patients presenting poor 24-h RAIU, particularly those with 24-h RAIU below 46.31%.

## Introduction

Graves’ disease (GD), an autoimmune disorder, is the most common cause of hyperthyroidism in iodine-sufficient areas [1]. The annual incidence rate is approximately 20–30 cases per 1000000 individuals, with a prevalence of 3% in females and 0.5% in males around the world [1]. Its incidence peaks at 30–60 years old and it affects 0.25–1.09% of the Chinese Han population [2]. At present, it is recognized that radioactive iodine (^131^I) treatment (RIT) is an effective and safe therapy for hyperthyroidism control [3]. To avoid ^131^I-induced hypothyroidism, even though a high rate of recurrence, antithyroid drugs (ATDs) were used in the past as the first therapy for GD in China. Currently, RIT is believed an effective intervention for GD treatment in China and has been widely accepted by doctors and patients [4]. The ^131^I treatment is thought to be not only affordable and safe, but also simple to use among Chinese patients. It is also a preferred choice for those who remain hyperthyroid after drug treatment [5].

Previous works have shown multiple predictive factors associated with the therapeutic outcome of GD, such as age, gender, pre-RIT serum levels of TSH or serum-free thyroxine (FT4), treatment with ATDs, thyroid gland mass, withdrawal of ATDs prior to RIT, and thyroid mass etc. [6–15]. Some scholars claimed that lower treatment success rates occurred in patients with high free T3 concentration, ophthalmopathy at presentation [16], higher 2-h radioactive iodine uptake (RAIU) [17], lower RIT dose, ^99m^Tc sodium pertechnetate thyroid uptake > 20.9%, and marked goiter [6,18], young male patients, and more severe cases of hyperthyroidism [7]. However, there is controversy regarding the most effective dose of iodide-131 (^131^I) in treatment of Graves’ hyperthyroidism. Different RAI dose regimens are used, including low dose, doses calculated based on the thyroid volume (TV) and complex calculations, fixed-dose protocol (185 MBq [5.0 mCi], 370 MBq [10.0 mCi] and 555 MBq [15.0 mCi]). The standard approach to ^131^I therapy has not been well established. Dose calculation of ^131^I aims to optimize the outcome of the treatment and minimize the residue radiation dose [19]. In the severe Graves’ hyperthyroidism, increasing radioiodine dose cannot improve cure rates [20], and thereby individualized dosimetry based on clinical or imaging techniques has been developed for calculation of RAI doses [21]. Nevertheless, there are still issues regarding administration of ^131^I activity individualized dosimetry, such as patient selection (in particularly those with ophthalmopathy), prescription algorithms, and the need for adjuvant thyrostatic medication [21–23]. The calculated-dose methods showed similar rates of amelioration and hypothyroidism with the cost-saving fixed-dose approach [8,24], there has been limited evidence found in China. Besides, it is still crucial to explore factors associated with treatment failure, regardless which regimen is adopted.

In the present study, we aim to investigate the factors predicting the outcomes of RIT with a calculated-dose administration for treatment of GD by retrospective review of 45 patients’ records before and after RIT.

## Materials and methods

### Patients

The present study was approved by the Institutional Review Board (IRB) of our institute. All experiments carried out were in accordance with the World Medical Association Declaration of Helsinki; all subjects provided the informed consent. We collected and evaluated the data of 45 hyperthyroid patients who had not been previously treated with ^131^I due to GD, followed in the Endocrinology Division from August 2015 to August 2016.

The inclusion criteria for GD patients were as follows: diffuse goiter, suppressed serum thyrotropin levels (TSH; reference values or RV = 0.35–4.94 µIU/ml), and high serum-free thyroxine (FT4; RV = 9.01–19.5 pmol/l) and free triiodothyronine (FT3; RV = 2.63–5.70 pmol/l).

The exclusion criteria for patients were as follows: (1) age ≤ 12 years, (2) pregnant or nursing female patients, (3) with clinical evidence of ophthalmopathy, (4) with large and compressive goiters (≥150 g) or intrathoracic goiters, (5) with a history of thyroidectomy and (6) with thyroid nodules suspicious of malignancy.

Those patients who were initially treated with ATDs for at least 18 months but ended up with therapeutic failure were referred to the Nuclear Medicine Department for RIT with a calculated-dose administration of ^131^I based on the volume of the lobe (V). All patients were instructed to discontinue the medication 2–7 days prior to RIT.

RIT was considered successful when euthyroidism or hypothyroidism was achieved after definitive ATD discontinuation for 1 year, with a single dose of ^131^I. Patients who exhibited hypothyroidism within 6 months after RIT were followed up for at least another 6 months to exclude transient hypothyroidism.

Patients’ gender and age at diagnosis, serum levels of FT3, FT4 and TSH were recorded pre-RIT and 1 year after RIT.

### TV and weight estimation

The TV was estimated before RIT as previously described [25,26]. In brief, the size was measured independently by two radiologists using Ultrasound System (GE Logiq 400 Pro, GE Healthcare, Wauwatosa, WI) with a 10-MHz probe. Longitudinal and transverse scans were performed for measurement of the depth (d), width (w) and length (l) of each lobe. The volume (V) of the lobe was calculated based on the ellipsoid formula: V (cm^3^) = 0.479 × d × w × l (cm). The TV was the sum of both lobes. And the thyroid weight was estimated according to each volume.

### Thyroid RAIU

All patients underwent 3- and 24-h RAIU tests before RIT. Patients received a 185-KBq (5 µCi) dose of sodium iodide (^131^I-NaI) on an empty stomach, after a low-iodine diet for 15 days prior to RIT. Through a FH-458 Thyroid Uptake Testing System (Beijing Nuclear Instrument Factory, Beijing, China), radioiodine uptake was measured 3 and 24 h after administration as previously introduced [17].

### RIT

RAI doses were calculated based on the TV: 555 MBq (TV < 30 ml), 740 MBq (TV between 30 and 60 ml), 925 MBq (TV between 60 and 90 ml), or 1100 MBq (TV > 90 ml) of ^131^I. If patients had severe hyperthyroidism or exhibited a thyroid gland mass ≥ 30 g that could not finish the related examinations, they would undergo fixed doses of either 555 MBq (15.0 mCi) or 740 MBq (20.0 mCi) of ^131^I.

### Follow-up after RIT

Patients were followed up 1, 3, 6 and 12 months after radioiodine administration by complete recording, and physical and laboratory (thyroid hormones FT3, FT4, TSH, TPO antibody, TG antibody) examination. RIT was considered successful if there was clinical and laboratory evidence of stable euthyroidism or hypothyroidism in the absence of ATDs after 12 months of ^131^I therapy. RIT was considered failed if hyperthyroidism persisted or relapsed. Patients who acquired hypothyroidism in 6 months after RIT were followed up for an additional 6 months to exclude transient hypothyroidism.

### Statistical analysis

All continuous variables were presented as mean ± SD, and categorical variables were presented as counts. One-way ANOVA was used to compare continuous variables of baseline characteristics among groups. The receiver operator characteristic (ROC) curve was used to identify the best threshold to discriminate RIT success (euthyroidism and hypothyroidism) and failure (persistence of hyperthyroidism), as well as the cut-off value at which 24-h RAIU can discriminate success and failure. Linear regression analysis was performed to identify the RAI dosage associated with follow-up TPO and TG antibody levels. The statistical significance was set at *P*<0.05.

## Results

### Patients and outcomes after RIT

A total of 45 patients (5 males and 40 females) with an average age of 37.78 ± 10.33 were included in the present study. The median follow-up was 11.6 months (range: 6–12 months). These patients presented a mean value of 3-h RAIU of 34.92 ± 18.01% and 24-h RAIU of 59.28 ± 14.24%. The mean value of estimated thyroid gland volume was 78.27 ± 33.24 cm^3^. Administered therapeutic RAI doses varied from 555 MBq (15.0 mCi) to 1100 MBq (30.0 mCi).

**Figure 1:**
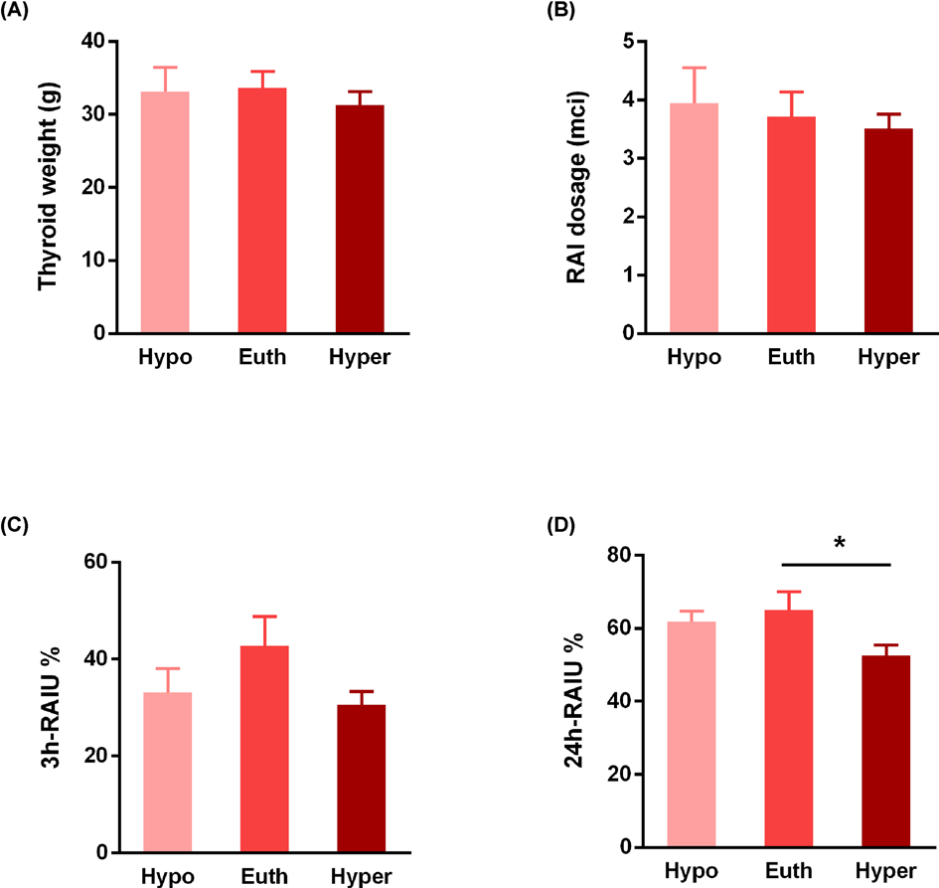
Only 24 h-RAIU was associated with the final outcome of RIT (**A**) Three groups with different outcomes (hypothyroidism or Hypo, euthyroidism or Euth, and hyperthyroidism or Hyper) had similar thyroid weights. (**B**) The RAI dosage did not affect the outcomes. (**C**) The 3 h-RAIU was similar among groups. (**D**) The 24 h-RAIU was associated with the final outcome of RIT, and this parameter in the Hyper group was significantly lower compared with Euth.

The baselines and clinical characteristics are listed in Table 1. After RIT, the remission rate was 62.2%, including 13 euthyroidism cases (28.9%) and 15 hypothyroidisms (33.3%). Among the patients who developed hypothyroidism after RIT, 9 (60%) were found within 6 months after RIT and 6 (40%) between 6 and 12 months after RIT. There were 17 patients (37.8%) remained hyperthyroid after RIT, among which 11 relapsed and 6 were controlled. No significant differences were found in FT3, FT4, TSH and thyroid weight among groups (Table 1 and Figure 1A).

**Table 1:** Baseline characteristics of patients according to post-RIT thyroid function

	Hypo	Euth	Hyper
Number	15	13	17
Age (years)	36.67 ± 10.23	40.07 ± 8.93	37.0 ± 11.64
Sex (female/male)	13/2	13/0	14/3
Pre-RIT FT3 (ng/dl)	21.88 ± 10.60	25.63 ± 16.12	24.45 ± 10.95
Pre-RIT FT4 (ng/dl)	52.14 ± 16.22	49.67 ± 19.97	44.45 ± 16.24
Pre-RIT TSH (μU/ml)	0.017 ± 0.045	0.037 ± 0.111	0.012 ± 0.019
RAI dose (mci)	3.94 ± 2.37	3.73 ± 1.58	3.51 ± 1.01

Data presented as Mean ± standard deviation. Three groups were divided according to the outcomes: hypothyroidism (Hypo), euthyroidism (Euth), and hyperthyroidism (Hyper).

### Effects of variables on post-RIT outcomes

**Figure 2:**
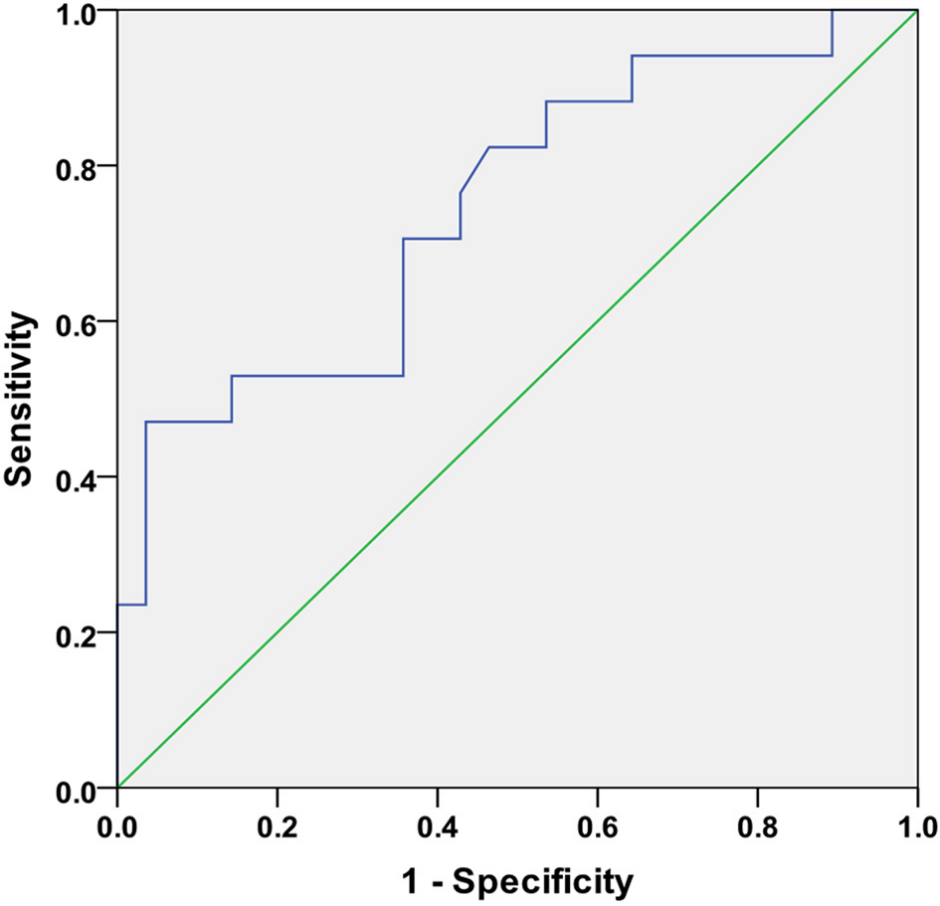
ROC curves used to identify the cut-off value of 24-h RAIU related to RIT success The 24-h uptake (AUC = 0.747) with a threshold of 46.31% (47.1% sensitivity and 96.4% specificity) could be referred for treatment success. A higher 24 h-RAIU value implied a higher probability of success especially when up to 46.31%.

First, we found no effects of RAI dosage on the outcome types, that patients with three types of ending had received similar levels of RAI dosages (Figure 1B). There are significant variations of the 24-h uptake (F_2,42_ = 3.491, *P*<0.05) among groups. The hyperthyroidism group exhibited a significantly lower 24h-RAIU compared with the euthyroidism group (Figure 1D, *P*<0.05). Whereas, we found no statistically significant differences in the following parameters: age (*P*=0.527), pre-RIT serum levels of FT3, FT4, TSH and 3-h RAI uptake (Figure 1C). This result suggested that 24h-RAIU might be useful in predicting the outcome of RIT at some extent. Next, we analyzed the 24-h uptake using the ROC curve to discriminate the success (euthyroidism or hypothyroidism) group from the failure (persistence of hyperthyroidism). As shown in Figure 2, we found the 24-h uptake (AUC = 0.747) with a threshold of 46.31% (47.1% sensitivity and 96.4% specificity) could be referred for treatment success. A higher 24h-RAIU value implied a higher probability of success especially when up to 46.31%. This is theoretically consistent with the known knowledge that a higher RAIU means more ^131^I enters the thyroid gland and results in more radiation to the gland and thus more successful therapy.

During our follow-up analysis, we found an interesting phenomenon, that higher RAI dosages may not necessarily benefit the patients, for no differences in RAI dosage were found between groups, instead, the RAI dosage significantly correlates with the TPO and TG antibody expression approximately 1 year later. The TPO and TG antibody levels exhibited a positive linear relationship with the RAI dosage (TPO Ab and dosage: F_1,43_ = 6.213, *P*<0.05, R square = 0.126; TG Ab and dosage: F_1,43_ = 14.12, *P*<0.001, R square = 0.247). However, the linear correlation was only found in the total sample set, and no differences were observed among three groups in expression of these two antibodies. Moreover, this correlation was majorly contributed by a patient with an extremely high RAI dosage, TPO antibody level (1000 U) and TG antibody level (1000 U). When this value was excluded, no positive correlation was found (*P*>0.05 for both TG Ab and TPO Ab). Therefore, this conclusion needs more evidence to support. However, Given TPO and TG antibodies are related with Hashimoto’s thyroiditis, and their expression suggest an abnormal autoimmunity. These results implied that exorbitant RAI dosages may not bring therapeutic benefits but induce additional disorders in GD, and the dosage could be chosen as low as possible when sufficient radiation has been reached.

## Discussion

The present study shows that a calculated dose of RAI based on TV was efficient for the treatment of GD. Besides, the 24-h RAIU may be an important reference to predict the therapeutic outcomes in this approach. An exorbitant RAI dosage may not benefit but induce extra TPO and TG antibody expression.

Radioiodine has been used for 60 years in treatment of autoimmune hyperthyroidism. We, here have shown that 24-h RAIU above 46.31% was a predictive factor for treatment success. In contrast with several other reports [6–14], we found that previous reported parameters, such as age, gender, pre-RIT serum levels of FT3, FT4 or TSH were not effective predictive factors for treatment outcome, suggesting that high dose RIT or fixed-dose RIT has no advantages over the calculated-dose method based on clinical and laboratorial parameters. The predictive role of 24 h-RAIU in RIT can be supported by some published works. For example, it had been pointed out that the 5h/24h-RAIU was positively related to treatment failure [29], which indirectly sound that higher 24 h-RAIU implies higher probability of success. Another intriguing result is that the TPO and TG antibody expression months later are positively correlated with the RAI dosage. It is agreed that high TG Ab or TPO Ab levels were considered to be at risk of thyroid cancer and thyroid autoimmunity [28,30,32]. However, this finding needs more supportive data, and it is early to tell that TG or TPO Ab variation is a necessary consequence of exorbitant RAI dosage.

The main limitation of the present study is the lack of data on thyroid autoantibodies titers, especially TRAb levels. Although TRAb does not represent a highly accurate indicator of disease remission, it may be of help in predicting disease severity and likelihood of treatment failure. However, it was not available as a routine laboratory assessment during the time of data collection. Because of the small number of participants in the present study, this indicator could not be acquired for majority of patients. Furthermore, it should be noted that the ROC curve for 24-h RAIU had a relatively low area under the curve (0.747), indicating the cut-off value of 46.31% may have insufficient discriminatory accuracy. Further study with large population is required to improve this cut-off value. Moreover, the overall successful rate of our RIT was not satisfactory (62.2%). Many known studies have reported the remission rate above 70% [29]. But there are still some early data showing a remission rate below 60% [27,31]. Our further study would focus on more factors which may limit this rate in our treatment process.

In conclusion, our study has shown that a calculated dose for treatment of GD is effective, but high failure rates are expected in patients presenting poor 24-h RAIU, particularly those with 24-h RAIU below 46.31%.

## Abbreviations

ATD: antithyroid drug
FT3: free triiodothyronine
FT4: serum-free thyroxine
GD: Graves’ disease
IRB: Institutional Review Board
RAIU: radioactive iodine uptake
RIT: radioactive iodine (^131^I) treatment
ROC: receiver operator characteristic
RV: reference value
TGAb: antithyroglobulin antibody
TPOAb: thyroid peroxidase antibody
TRAb: thyrotrophin receptor antibody
TSH: thyroid stimulating hormone
TV: thyroid volume
